# Pancreatic B-cell function is altered by oxidative stress induced by acute hyperglycaemia

**DOI:** 10.1111/j.1464-5491.2007.02058.x

**Published:** 2007-02

**Authors:** Y Miyazaki, H Kawano, T Yoshida, S Miyamoto, J Hokamaki, Y Nagayoshi, H Yamabe, H Nakamura, J Yodoi, H Ogawa

**Affiliations:** Department of Cardiovascular Medicine, Graduate School of Medical Sciences, Kumamoto UniversityKumamoto; * Thioredoxin Project, Department of Experimental Therapeutics, Translational Research Centre, Kyoto University HospitalKyoto, Japan; † Department of Biological Responses, Institute for Virus Research, Kyoto UniversityKyoto, Japan

**Keywords:** blood glucose, impaired glucose tolerance, oxidative stress, postprandial hyperglycaemia

## Abstract

**Aims:**

Type 2 diabetes is preceded by a symptom-free period of impaired glucose tolerance (IGT). Pancreatic B-cell function decreases as glucose intolerance develops. In many patients with IGT, fasting blood glucose is within normal limits and hyperglycaemia occurs only postprandially. We examined whether pancreatic B-cell function changes during acute hyperglycaemia induced by oral glucose loading.

**Methods:**

We calculated the insulinogenic index (I.I.) as an indicator of pancreatic B-cell function and measured serum levels of thioredoxin, a marker of cellular redox state, and 8-hydroxy-2′-deoxyguanosine (8-OHdG), a marker of oxidative stress, during a 75-g oral glucose tolerance test (OGTT) in 45 subjects [24 patients with normal glucose tolerance (NGT), 14 with IGT and seven with Type 2 diabetes].

**Results:**

Thioredoxin levels decreased after glucose loading [66.1 ± 23.7, *59.3 ± 22.4, *49.3 ± 21.2 and *37.7 ± 18.0 ng/mL, fasting (0 min) and at 30, 60 and 120 min, respectively; *P < 0.001 vs. fasting]. In contrast, concentrations of 8-OHdG peaked at 30 min and then gradually decreased (0.402 ± 0.123, *0.440 ± 0.120, †0.362 ± 0.119 and †0.355 ± 0.131 ng/mL, *P < 0.05 vs. fasting, †*P <* 0.01 vs. 30 min). The insulinogenic index correlated with the change in thioredoxin levels (*r =* 0.34, *P* < 0.05). However, there was no relationship with the change in 8-OHdG levels from 0 to 30 min.

**Conclusions:**

Hyperglycaemia in response to oral glucose impairs pancreatic B-cell function with decreasing thioredoxin levels. The augmented oxidative stress induced by hyperglycaemia may affect the cellular redox state. These findings strongly suggest that repeated postprandial hyperglycaemia may play an important role in the development and progression of diabetes mellitus.

## Introduction

Diabetes mellitus is associated with the development of atherosclerosis [1]. Both macrovascular disease, such as acute coronary syndrome, stroke and claudication, and microvascular disease, such as diabetic nephropathy and retinopathy, are commoner in diabetic than in non-diabetic populations and contribute to the morbidity and mortality associated with diabetes [1–3]. The predominant clinical form is Type 2 diabetes, which accounts for > 90% of all cases [2,3]. Most Type 2 diabetes is preceded by a symptom-free period of impaired glucose tolerance (IGT), characterized by a response to oral glucose loading that is abnormal but does not satisfy the criteria for diabetes [1]. Patients with Type 2 diabetes show failure of glucose-induced insulin secretion, which is characterized by a decrease in the first phase of glucose-induced insulin secretion, delayed hyperinsulinaemia and, latterly, failure of insulin synthesis. Since Type 2 diabetes has a polygenetic background, several factors may cause the failure of B cells. One possibility is several types of stress, such as oxidative stress. Recently, we and other investigators reported that acute hyperglycaemia induces oxygen-derived free radicals [4–7], which are involved in pancreatic B-cell dysfunction and apoptosis in an animal model of Type 1 diabetes [8].

The redox state is finely tuned to preserve cellular homeostasis through the expression and regulation of oxidant and antioxidant enzymes. Mammalian cells have a complex network of antioxidants such as catalase, superoxide dismutase and glutathione peroxide to scavenge reactive oxygen species. In addition to these enzymes, the members of the thiol-disulphide oxidoreductase family act as cytoprotective antioxidants [9]. One of the most important thiol-disulphide oxidoreductases is thioredoxin (TRX) [10].

The purpose of the present study was to examine the effect of acute hyperglycaemia induced by oral glucose loading on pancreatic B-cell function. We further addressed the role of oxidative stress in pancreatic B-cell function during oral glucose loading.

## Patients and methods

### Study subjects

The study group comprised 45 subjects (mean age 67 ± 11 years) who were admitted to our hospital for investigation of coronary artery disease. None was known previously to have diabetes. Fasting glucose levels were < 7.0 mmol/l and a 75-g oral glucose tolerance test (OGTT) was done to examine risk factors for coronary artery disease. Diabetes was diagnosed according to World Health Organization criteria [2]. Twenty-four patients had normal glucose tolerance (NGT), 14 had IGT and seven had Type 2 diabetes mellitus (DM). Fourteen patients in the NGT group, eight in the IGT group and five in the diabetes group had ischaemic heart disease. The characteristics of the patients are shown in Table 1. Written informed consent was obtained from each patient before the study was commenced. The procedures used in the study were approved by the ethics committee at our institution.

**Table 1:** Characteristics of study subjects

	NGT (*n* = 24)	IGT (*n* = 14)	DM (*n* = 7)	*P*-value
Age (years)	68 ± 12	64 ± 11	72 ± 3	NS
Men/women (*n*)	10/14	10/4	2/5	NS
BMI (kg/m^2^)	23.7 ± 3.7	24.8 ± 4.7	25.7 ± 7.4	NS
HbA_1c_ (%)	5.6 ± 0.4	5.8 ± 0.4	6.5 ± 0.5	*<* 0.01
FBG (mmol/l)	4.9 ± 0.4	5.4 ± 0.8	6.2 ± 0.7	*<* 0.01
SBP (mmHg)	133 ± 25	135 ± 25	135 ± 24	NS
DBP (mmHg)	79 ± 18	80 ± 12	70 ± 15	NS
Smoker/non-smoker	3/21	2/12	2/5	NS
Total cholesterol (mmol/l)	5.0 ± 0.90	5.5 ± 0.70	5.3 ± 0.62	NS
HDL-cholesterol (mmol/l)	1.29 ± 0.34	1.42 ± 0.47	1.55 ± 0.62	NS
Triglyceride (mmol/l)	3.2 ± 1.53	4.2 ± 2.02	3.1 ± 1.42	NS

BMI, Body mass index; DBP, diastolic blood pressure; DM, diabetes mellitus; FBG, fasting blood glucose; HDL, high-density lipoprotein; IGT, impaired glucose tolerance; NGT, normal glucose tolerance; SBP, systolic blood pressure.

### Study design

The study was performed during a 75-g OGTT after a 12–14-h fast. Blood samples were obtained in the fasting state, and 30, 60 and 120 min after the administration of a 75-g glucose equivalent load (Trelan-G, Takeda, Japan). The plasma glucose concentration was determined with an autoanalyser using a glucose oxidase method. The serum insulin concentration was measured by immunoradiometric assay using an antihuman insulin antibody. Fasting serum total cholesterol and triglyceride concentrations were measured enzymatically, and the serum high-density lipoprotein cholesterol concentration was measured by heparin-Ca^2+^/Ni^2+^ precipitation [7,11]. The insulinogenic index (I.I.) was calculated as the ratio of the increment of insulin to that of plasma glucose 30 min after the glucose load [(30 min insulin − fasting insulin)/(30 min glucose − fasting glucose)][12,13].

Adaptation to stress evokes a variety of biological responses in humans. TRX is an important constituent of cellular antioxidant buffering systems that control the redox state of proteins, which is released into the extracellular space [14]. We thus measured the serum levels of TRX as a marker of the cytoprotective antioxidant system (ELISA kit; Fuji Rebio, Tokyo, Japan) [14–17]. The detection limit of TRX was 2.0 ng/mL and the intra- and interassay coefficients were 0.81–3.74% and 4.87–6.97%, respectively [14]. Plasma levels of 8-hydroxy-2′-deoxyguanosine (8-OHdG), a sensitive marker of oxidative stress, were measured with enzyme-linked immunosorbent assay (ELISA) (8-OHdG check; Japan Institute for the Control of Ageing, Fukuorio, Shizuoka).

### Statistical analysis

Comparisons of data between the three groups were performed using one-way analysis of variance (anova) followed by Bonferroni's multiple comparison test. The χ^2^ test was used to compare gender and the prevalence of smokers. Changes in variables were assessed by two-way anova with repeated measures followed by post hoc testing with Scheffe's test. Correlations between the insulinogenic index and plasma TRX concentrations, insulinogenic index and plasma 8-OHdG concentrations were examined with linear regression analysis. Statistical significance was defined as *P* < 0.05.

## Results

There were no differences in serum concentrations of total cholesterol, low-density lipoprotein cholesterol, triglyceride, age or blood pressure between the three groups (Table 1). The differences in body mass index were not significant.

**FIGURE 1:**
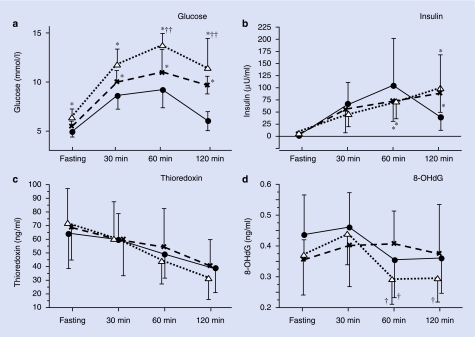
Blood glucose, insulin, thioredoxin and 8-hydroxy-2′-deoxyguanosine (8-OHdG) concentrations during an oral glucose tolerance test in subjects with normal glucose tolerance (NGT, •), impaired glucose tolerance (IGT, ×) and diabetes (Δ). *P < 0.01 vs. NGT; †*P <* 0.05 vs. IGT; ††*P <* 0.01 vs. IGT.

Figure 1 and Table 2 show the plasma glucose and serum insulin concentrations during the OGTT in the three groups. The increase in fasting plasma glucose levels as glucose tolerance deteriorated from IGT to DM was not significant, but the difference between the DM group and the NGT group did reach statistical significance (*P <* 0.01). After the glucose load, both the plasma glucose and the serum insulin concentrations increased in each group. Plasma glucose levels were significantly higher in the IGT and the DM groups than in the NGT group at 30, 60 and 120 min after glucose loading (*P <* 0.01). The concentrations were not different between the IGT group and the DM group at 30 min, but were significantly higher in the DM group than in the IGT group at both 60 and 120 min (*P <* 0.01). Fasting serum insulin concentrations were not significantly different between the three groups and increased significantly in each group after the glucose load. In the NGT group, serum insulin concentrations peaked at 60 min, and then decreased. In contrast, they continued to increase until 120 min after the glucose load in the IGT and DM groups. Serum insulin concentrations at 60 min in the NGT group were the highest in the three groups (*P <* 0.01) and at 120 min were the lowest (*P <* 0.01). There was no difference in the insulin concentrations between the IGT and DM groups at 60 min or 120 min. Serum concentrations of TRX gradually decreased after oral glucose loading in each group (*P <* 0.05 vs. fasting; Fig. 1). Concentrations of 8-OHdG were similar in all three groups, both fasting and at 30 min, and did not change from fasting to 30 min in any group. 8-OHdG concentrations decreased in the NGT and DM groups from 30 to 60 min (*P <* 0.01). There were no significant changes in the IGT group.

**Table 2:** Concentrations of measured variables during oral glucose tolerance test

		Fasting	30 min	60 min	120 min
Glucose (mmol/l)	NGT	4.9 ± 0.4	8.6 ± 1.4**	5.9 ± 1.0**	4.3 ± 1.8
	IGT	5.4 ± 0.4	9.9 ± 1.6**	10.9 ± 2.3**	9.6 ± 0.8**
	DM	6.2 ± 0.7	11.6 ± 1.6**	13.5 ± 1.3**	11.2 ± 3.1**
Insulin (µU/mL)	NGT	6.1 ± 2.2	65.1 ± 45.4**	105.2 ± 93.1**	42.3 ± 26.5**
	IGT	7.9 ± 4.5	58.3 ± 46.8**	73.7 ± 39.1**	89.1 ± 37.6**
	DM	11.1 ± 5.6	44.3 ± 20.9**	68.2 ± 28.2**	98.3 ± 67.2**
Thioredoxin (ng/mL)	NGT	64.0 ± 19.9	59.1 ± 19.2*	48.5 ± 17.4**	38.7 ± 17.8**
	IGT	67.9 ± 29.8	59.6 ± 26.3*	53.6 ± 28.5**	39.7 ± 19.9**
	DM	70.0 ± 19.9	59.5 ± 27.6*	43.4 ± 16.8**	30.4 ± 14.6**
8-OHdG (ng/mL)	NGT	0.44 ± 0.13	0.46 ± 0.11	0.35 ± 0.13**	0.36 ± 0.11**
	IGT	0.35 ± 0.11	0.40 ± 0.13*	0.41 ± 0.11*	0.37 ± 0.16
	DM	0.37 ± 0.07	0.44 ± 0.09*	0.28 ± 0.07**	0.29 ± 0.07**

* *P* < 0.05 vs. fasting;

** *P* < 0.01 vs. fasting.

DM, Diabetes mellitus; IGT, impaired glucose tolerance; NGT, normal glucose tolerance.

**FIGURE 2:**
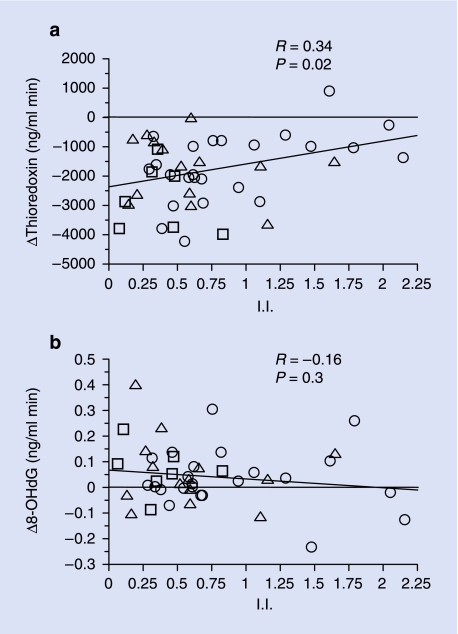
Correlation between insulinogenic index and the change in thioredoxin from 0 to 30 min, and the relation between the insulinogenic index and the change in 8-hydroxy-2′-deoxyguanosine (8-OHdG) from 0 to 30 min. I.I., Insulinogenic index; Δthioredoxin, the change in thioredoxin; Δ8-OHdG, the change in 8-OHdG. ○, Normal glucose tolerance; Δ, impaired glucose tolerance; □, diabetes.

The insulinogenic index was highest in the NGT group and lowest in the DM group (NGT 0.904 ± 0.511, IGT 0.605 ± 0.435, DM 0.376 ± 0.256; *P* = 0.02 NGT vs. DM). In the combined cohort, the insulinogenic index correlated with the change in TRX during oral glucose loading (ΔTRX) (*r =* 0.34, *P* < 0.05) (Fig. 2). However, there was no relationship with the change in 8-OHdG concentrations from fasting to 30 min (Δ8-OHdG).

## Discussion

There is widespread agreement that diabetes increases the risk of cardiovascular disease [2,3,18–21]. The mechanism of this increased risk is controversial [3,18–22], but hyperglycaemia may play an important role in patients with diabetes and IGT [22,23]. Although hyperglycaemia is clearly related to diabetic microvascular complications [22], its contribution to the increased risk of atherosclerosis in Type 2 diabetes remains controversial. Type 2 diabetes and IGT are commonly associated with other risk factors, such as dyslipidaemia, hypertension and obesity [3,18–22,24]. All of these factors may contribute to the occurrence of cardiovascular disease in patients with diabetes and IGT.

Failure of insulin secretion in patients with Type 2 diabetes and IGT is characterized by decreased first-phase glucose-induced insulin secretion, delayed hyperinsulinaemia and the late development of failure of insulin synthesis [25–29]. In the present study, the peak in the glucose concentration was 60 min after glucose loading in each group. In contrast, although the peak of insulin concentration was 60 min after glucose loading in the NGT group, the peak was at 120 min in the DM and IGT groups. These findings suggested that insulin secretion in response to oral glucose loading was already impaired in the DM and the IGT group.

In the present study, oral glucose loading decreased serum TRX concentrations. Thioredoxins are critical for redox regulation of protein function and signalling via thiol redox control [30]. TRX is induced by stress, and protects cells from various types of stress, such as viral infection. TRX is not only a scavenger of free reactive oxygen species but also regulates the activity of various intracellular molecules, including transcription factors such as nuclear factor-κB, activator protein 1, myb, redox factor 1 and mitogen-activated kinase [31]. Thus, cytosolic mammalian TRX has numerous functions in the defence against oxidative stress, control of growth and apoptosis [32]. TRX and the redox system it modulates have an important role in cellular defence against cytotoxicity caused by reactive oxygen species. In the present study, we could not show a relationship between the insulinogenic index and TRX or 8-OHdG concentrations. However, there was a correlation between the change in TRX concentrations after the oral glucose loading and the insulinogenic index (Fig. 2). The changes in glucose concentrations from fasting to 30 min after glucose loading are the largest changes after the glucose load. Thus, the concentrations of 8-OHdG increased 30 min after glucose loading, and increased oxidative stress may affect the TRX concentrations. The increased TRX is used to protect the cell against oxidative stress. Therefore, both released extracellular TRX and 8-OHdG concentrations decrease 60 min after glucose loading [32]. The present study demonstrates that hyperglycaemia after a glucose load may affect the cellular antioxidant system in humans.

Glucose is the primary fuel and regulator of pancreatic islet B-cell function. The primary function of insulin is to maintain blood glucose concentrations in the normal range. However, chronic hyperglycaemia impairs glucose-induced insulin secretion and insulin gene expression [33]. One of the potential mechanisms is oxidative stress, because glucose is able to generate reactive oxygen species [4–7], which have adverse effects on islet function [34–37]. The augmented reactive oxygen species generation produced by exposure to elevated glucose may play an important role in the diminished activity of B-cells [38,39]. Although the pathogenesis of Type 2 diabetes is multifactorial, B-cell functional abnormalities are present at a very early stage of development of the disease [40,41]. It is well known that B-cell dysfunction is observed even in patients with IGT, as shown in the present study. Our findings suggest that increased reactive oxygen species induced by postprandial hyperglycaemia may affect B-cell function. Consequently, insulin secretion in response to blood glucose may become impaired. In the present study, 8-OHdG, a marker of oxidative stress, increased after glucose loading, but TRX, a marker of cellular redox state, decreased. There was a significant relationship between TRX concentrations and insulinogenic index, a marker of insulin secretory activity of B-cells. Thus, the augmented reactive oxygen species production may reduce cellular antioxidant defences. This may affect the B-cell and result in impairment of insulin secretion.

A variety of mechanisms may generate reactive oxygen species during acute hyperglycaemia [42–44]. These include autoxidation, non-enzymatic glycation of proteins due to extended exposure to hyperglycaemia, metabolism of glucose via aldose reductase with changes in sorbitol-myoinositol concentrations and the increased *de novo* synthesis of diacylglycerol from glycolytic intermediates and subsequent activation of the protein kinase C pathway [43,44]. However, non-enzymatic glycation processes do not account for the rapid increase of oxidative products in response to acute hyperglycaemia, because glycation processes occur slowly over days to weeks [45].

Our results suggest that acute hyperglycaemia produces reactive oxygen species, and that the increase in reactive oxygen species affects cellular antioxidant defences. In animal models, antioxidant treatment protects against the onset of diabetes [46]. In addition, acarbose effectively reduced the risk of development of diabetes in patients with IGT [47]. Since this α-glucosidase inhibitor reduces postprandial hyperglycaemia, protection of B-cells from reactive oxygen species after postprandial hyperglycaemia could be a possible mechanism by which acarbose prevents progression to diabetes mellitus. These previous reports support our findings.

It is possible that TRX is induced by hyperinsulinaemia or dyslipidaemia in IGT or diabetes and thus alters the insulinogenic index. Thus, further studies are needed to clarify the role of hyperinsulinaemia and dyslipidaemia in insulin secretion in humans.

In conclusion, acute hyperglycaemia in response to oral glucose loading generates reactive oxygen species, which may affect the cellular redox state. Thus, postprandial hyperglycaemia reduces pancreatic B-cell function, and results in impairment of insulin secretion. These findings strongly suggest that repeated postprandial hyperglycaemia may play an important role in the development and progression of diabetes mellitus.

## Conflict of interest

None to declare.

## Abbreviations

8-OHdG: 8-hydroxy-2′-deoxyguanosine
anova: analysis of variance
DM: diabetes mellitus
IGT: impaired glucose tolerance
I.I.: insulinogenic index
NGT: normal glucose tolerance
OGTT: oral glucose tolerance test
TRX: thioredoxin
